# Paradoxical effects of altruism on efforts to mitigate climate change

**DOI:** 10.1038/s41598-022-17535-y

**Published:** 2022-07-29

**Authors:** A. Fossas-Tenas, B. W. Ibelings, J. Kasparian, J. Krishnakumar, J. Laurent-Lucchetti

**Affiliations:** 1 Institute for Environmental Sciences, University of Geneva, Bd Carl Vogt 66, 1211 Geneva 4, Switzerland; 2 Department F.-A. Forel for Environmental and Aquatic Sciences, University of Geneva, Bd Carl Vogt 66, 1211 Geneva 4, Switzerland; 3 Group of Applied Physics, University of Geneva, Rue de l’École de Médecine 20, 1205 Geneva, Switzerland; 4 Institute of Economics and Econometrics, GSEM, University of Geneva, Uni Mail, 1211 Geneva 4, Switzerland

**Keywords:** Environmental economics, Environmental social sciences

## Abstract

It is common wisdom that altruism is a crucial element in addressing climate change and other public good issues. If individuals care about the welfare of others (including future generations) they can be expected to unilaterally adapt their behaviour to preserve the common good thus enhancing the wellbeing of all. We introduce a network game model featuring both altruism and a public good (e.g. climate) whose degradation affects all players. As expected, in an idealistic fully connected society where all players care about each other, increasing altruism results in a better protection of the public good. However, in more realistic networks where people are not all related to each other, we highlight an intrinsic trade-off between the effects of altruism on reducing inequality and the preservation of a global public good: the consumption redistribution generated by a higher altruism is partly achieved by lowering income transfers towards protection of the public good. Therefore, it increases overall consumption and is thereby detrimental to the public good. These results suggest that altruism, although good from a welfarist point of view, is not in itself sufficient to simultaneously solve public good and inequality issues.

## Introduction

During the last decades a global increase in the world income has been associated with a sharp deterioration of the environment, e.g. as climate change^1^, biodiversity loss^2^ or worldwide pollution, and an increase in within-country inequalities^3^. It is often considered that promoting altruism, through education or social programs, will help to solve these fundamental problems. Human altruism, usually defined as the ability to make sacrifices to the benefit of others without expecting a personal reward, is known to be a powerful element of cooperation^4^. Therefore, it is reasonable to infer that fostering altruism will promote individual efforts to preserve the environment. This claim is further motivated by empirical data showing that altruism correlates with pro-environmental behaviour^5,6^.

**Figure 1:**
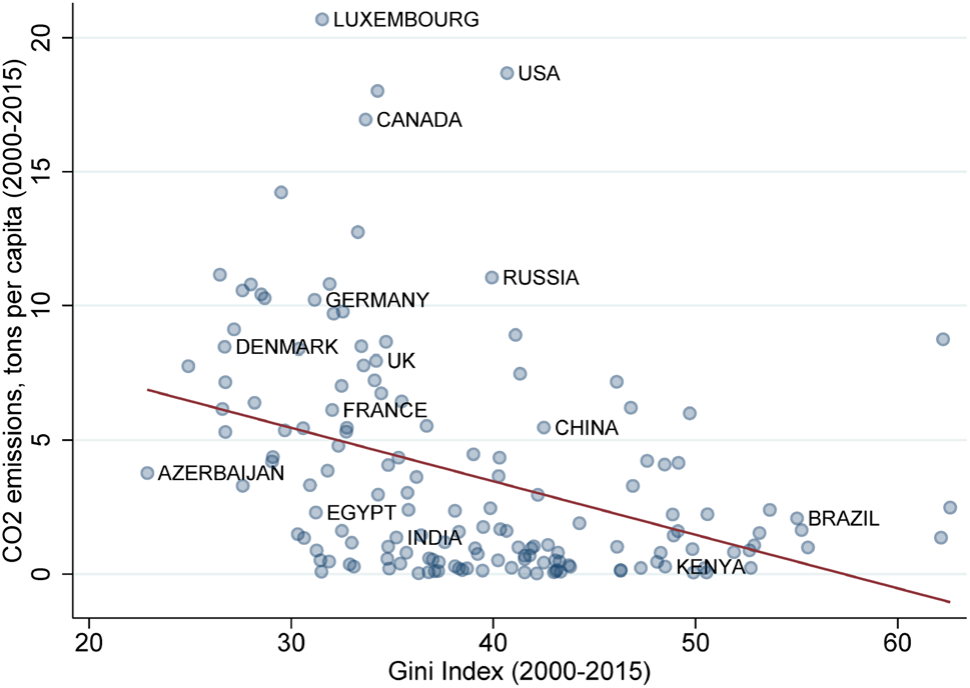
Average per capita $CO_2$ emissions produced in each country over the 2000–2015 period (source: world bank data) and “income inequalities”, measured using the well-known GINI coefficient of each country, averaged over the period 2000–2015 (source: World Wealth and Income Database). The figure displays a negative correlation $(-0.39)$ for 153 countries over the period.

However, this common wisdom is seemingly at odds with empirical evidence pointing at a puzzling negative correlation between income inequality and climate emissions. For example, countries with low income Gini coefficients—the most commonly used income inequality index—tend to feature high per-capita carbon emissions over the period 2000–2015^7^ (data shown in Fig. 1). In particular, this negative cross-country relation appears to be robust to a battery of controls^8^ (such as GDP, population, energy consumption etc) while the within-country result—accounting for fixed effects—is less robust. This suggests that the cross country relation is likely driven by some unobserved time-invariant country characteristics, such as the institutional setup or cultural values (for example altruism).

This observation, while non-causal, casts a doubt on the widely held belief that countries with a more equal distribution of income are also more likely to efficiently reduce their carbon footprint. As countries with low income inequalities also tend to be the ones with a high average level of altruism in the population^9,10^, altruism might in fact be insufficient, or even detrimental, to tackle environmental issues. Andreoni^11^ indeed finds that altruism is insufficient to explain the extensive and intensive provision of public goods.

Given the commonly perceived positive role of altruism in solving both inequality and environmental issues, it is critically important to address and better understand a potential trade-off between the two. Here we use game theory to investigate the hidden conflict on preservation of a global common good (climate) with the reduction of inequality due to changes of the altruism parameter in a network economic model based on Bramoullé, Bourlès and Perez-Richet^12^.

## Results

In this section, we first describe our model and show how the functional forms give an explicit equation for the social optimum as well as the Nash equilibrium equations. We then describe in more details the behaviour of two specific networks types: complete networks and regular trees, based on numerical simulations.

### Analytical results

#### Model description

Game theory is a widespread tool for modeling social interaction in economics since von Neumann^13^. In the game considered here, the players are initially endowed with a personal income $w_i$ and they are part of a network defined by their fixed bilateral altruistic bonds (as in Bourlès, Bramoullé and Perez-Richet^12^). Furthermore, they all benefit (or suffer) from the *quality* ${\mathscr {G}}$ of a global public good (e.g. the climate, air, water quality, food resource, etc) that depletes with total consumption, $C=\sum _{i=1}^N {c_i}$.

Players aim at maximizing their *social utility*, $u_i$, which is a function depending of personal consumption, $c_i$, the sum of the consumptions of the players they share an altruistic bond with (hereafter called *i*’s *neighbours*) and on the quality of the public good ${\mathscr {G}}$ in the following way:

1 $$\begin{aligned} u_{i} (c_1,\ldots ,c_N,W,S_0) = \left( \sqrt{c_i} +\alpha \sum _{j \in {\mathscr {N}}(i)} \sqrt{c_j}\right) \cdot {\mathscr {G}} \end{aligned}$$

where $c_i$ denotes final consumption of player *i*, ${\mathscr {N}}(i)$ denotes *i*’s neighbours, $\alpha$ ($0 \le \alpha <1$) is the strength of all altruistic bonds and *W* is the sum of all personal incomes. We use a Cobb-Douglas utility function because of its well-known analytical tractability and because it embeds a degree of complementarity between consumption and the quality of the public good. This desirable property would disappear if we were considering public good quality as an additive term (i.e. the case in which the public good is modelled as a passive player connected to all players and only receiving transfers).

To do so, they have to decide how to partition these initial incomes into personal consumption, transfers to neighbours, and transfers for the improvement of the public good state, which benefit all players. The quality of the public good is defined in our model as

2 $$\begin{aligned} {\mathscr {G}}(S_0,W,T) = \sqrt{S_0 + T - C^2/2}, \end{aligned}$$

where $S_0$ denotes the initial state of the public good, $C = \Sigma c_i$ is the overall consumption of players, $T=W-C$ is the total sum of transfers from players to the public good, and *W* is the (total) initial income.

More precisely, let $t_{i,j}$ denote the income transfer from player *i* to player *j* and $t_{i,S}$ the income transfer from player *i* to the public good. Then,

3 $$\begin{aligned} c_i = w_i - t_{i,S} + \sum _{j \in {\mathscr {N}}(i)} \left( t_{j,i} - t_{i,j} \right) . \end{aligned}$$

Furthermore, the total budget has to be balanced, meaning that all of it is either consumed (by agent *i* or by her neighbours receiving the transfer) or transferred to the public good:

4 $$\begin{aligned} \sum _{i=0}^{N-1} w_i = \sum _{i=0}^{N-1} c_i + \sum _{i=0}^{N-1}\sum _{j=0}^{N-1} t_{i,j} + \sum _{i=0}^{N-1} t_{i,S}, \end{aligned}$$

where *N* denotes the number of players and $t_{i,i}=0$.

Note that the consumption inequality can be reduced with respect to the initial income inequality either through private transfers between neighbours (income redistribution) or via direct transfers to the public good. Both mechanisms can take place simultaneously, the crucial difference between the two being that private transfers only benefit the direct recipients, while transfers to the public good benefit all players in the network through the public good quality factor ${\mathscr {G}}$ of Eq. (2).

#### Social optimum

From the point of view of an external observer of the game—a *social planner*—given a total amount of income *W* and an initial state of the public good $S_0$, there exists a socially optimal amount of consumption

5 $$\begin{aligned} C^\star = \frac{-2 + \sqrt{4+6(S_0+W)}}{3}, \end{aligned}$$

obtained by maximizing the sum of all players’ social utilities which can be written as

6 $$\begin{aligned} U_S(c_1,\ldots ,c_N,W,S_0)= & {} \sum _{i=1}^N u_{i} \\= & {} \left( \sum _{i=1}^N (1+ \alpha d_i) \sqrt{c_i}\right) \sqrt{S_0 + W - C -\frac{C^2}{2}}, \nonumber \end{aligned}$$

where *N* is the number of players, and $d_i$ the degree of the *i*-th player in the network (the number of connections involving player *i*). Since $U_S$ is a concave function of each $c_i$, it has a single maximum. This maximum is computed by finding the point at which all partial derivatives vanish (see Supplementary Material for the details of this calculation).

It is worth noting that $C^\star$ depends on the *total initial budget*, meaning players’ income plus initial state of the public good. Hence, $C^\star$ defines a boundary between two regimes: when $W > C^\star$, the negative impact of a degraded public good is so large that it requires the transfer of all wealth beyond $C^\star$ to the public good to stay at the social optimum (think about the need to adjust consumption in order to maintain $CO_2$ emissions below the threshold for uncontrolled global warming). However, when $W < C^\star$, the loss of total utility through public good damage is outweighed by the increase of the direct utility of consumption (think about the situation in the pre-industrial era). Therefore optimal consumption in this case equals *W*.

#### Nash equilibria equations

We are particularly interested in analyzing the decentralized model output, where all players maximize their own social utilities independently: the Nash equilibria. The social utility $u_i$ is a concave function, thus first-order equations are (necessary and) sufficient conditions for Nash equilibria. Let $W = \sum _{i=1}^N w_i$ be the total income, where $w_i$ is player *i*’s initial income. Let $S_0$ be the initial state of the public good, *N* the number of players, $c_i$ the personal consumption of player *i* and ${\mathscr {N}}(i)$ denote the set of player *i*’s neighbors.

At equilibrium, the marginal utility $\frac{\partial u_{i}}{\partial c_i}(c_1,\ldots ,c_N,W,S_0)$ of the own consumption of each player *i* is bounded by the marginal utility of their transfer $t_{i,k}$ to each of their neighbour *k*:

7 $$\begin{aligned} \frac{\partial u_i}{\partial c_i}(c_1,\ldots ,c_N,W,S_0) \ge \frac{\partial u_i}{\partial t_{i,k}}(c_1,\ldots ,c_N,W,S_0) \end{aligned}$$

and the equality holds when the transfer from player *i* to player *k* indeed occurs.

Similarly, the marginal utility of consumption for each player is bounded by the marginal utility of their transfer $t_{i,\text {S}}$ to the public good,

8 $$\begin{aligned} \frac{\partial u_i}{\partial c_i}(c_1,\ldots ,c_N,W,S_0) \ge \frac{\partial u_i}{\partial t_{i,\text {S}}}(c_1,\ldots ,c_N,W,S_0) \end{aligned}$$

and, again, the equality holds when a transfer from player *i* to the public good actually takes place.

Therefore, Nash equilibria are governed by two types of constraints (see the Suplementary Information for the details of the calculations). First, player *i* will transfer to player *k* only if sufficiently richer: i.e. if their consumption difference exceeds the ratio allowed by altruism

9 $$\begin{aligned} c_i \ge \alpha ^2 c_k \qquad \forall k \in {\mathscr {N}}(i). \end{aligned}$$

This condition is directly inherited from the variant of the model without a common good^12^ and highlights the potential directions that private income transfers have to follow to reduce consumption inequalities with respect to income inequalities.

The second condition relates the supplementary social utility provided by an increase in consumption, to the degradation of the public good caused by the same increase in consumption. Since both have to balance each other for each player individually, one has the following condition for Nash equilibria:

10 $$\begin{aligned} \frac{S_0 + W - C - \frac{C^2}{2}}{1+C} \ge \max _{i\le N}\left\{ c_i + \sum _{k \in {\mathscr {N}}(i)} \alpha \sqrt{c_i c_k}\right\} . \end{aligned}$$

The maximum function on the right-hand side of Eq. (10) implies that, except in case of huge disparities in the initial income distribution, players with the highest number of altruistic bonds will be more strongly impacted by the detrimental consequences of (higher) consumption on the public good quality. They will therefore reduce the most their consumption, and transfer the highest amount of personal income to the public good if the latter degrades. Similarly, among players having the same number of neighbours, condition (10) will first be violated for the richest players. They will hence reduce their consumption so as to transfer to the public good. Indeed, their relatively higher level of consumption together with the concavity of the utility function makes it easier for them to cut consumption for a higher public good quality. Note that in specific cases, Eq. 10 implies that consumption inequality could be larger than the initial income inequality. In particular, in a network where players have equal income but different numbers of neighbours, the most connected players will perceive more the detrimental effects of the public good degradation. They will therefore transfer more to the public good and enjoy a lower consumption than the less connected players.

### Results for specific network types

Our goal is to compare the decentralized solutions to the socially optimal one. While it is clear from Eq. (9) that increasing the altruism coefficient always reduces consumption inequality, its impact on consumption itself is less straightforward. We measure inequality with the Gini coefficient and we define *overconsumption* as the ratio $\frac{C}{C^\star }$ of actual consumption to its value at social optimum.

#### Complete network

**Figure 2:**
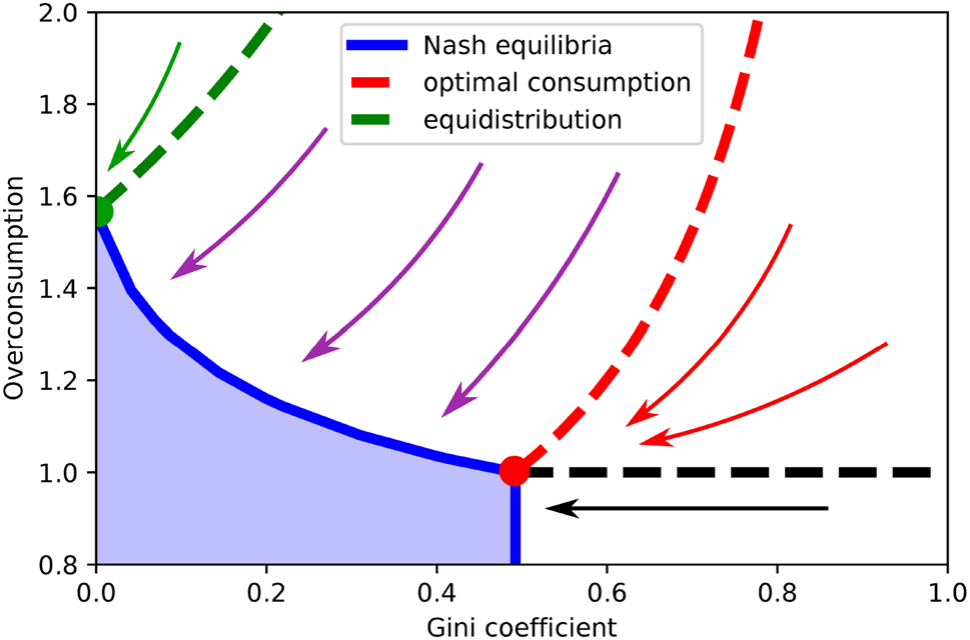
Phase diagram for a complete network of 100 players, among which a single rich one. In blue the set of Nash equilibria—for all players, to consume their initial income is optimal having considered the decisions of other players. All other initial conditions lead to a Nash equilibrium on the boundary, following the arrows (equidistributed consumption in green, no overconsumption in red).

A highly optimistic—while unrealistic, as discussed below—situation is the complete network case, where every pair of players is altruistically connected. Given a fixed amount of $S_0+W = 10^6$ and a complete network of 100 players with a single rich one, Fig. 2 displays the phase diagram of Nash equilibria in a plane defined by the Gini coefficient and the overconsumption ratio $C/C^\star$ (see Supplementary Information for the detailed equations and algorithm).

Our simulations identify which points are Nash equilibria and define the basins of attraction for each of these equilibria. In the blue region, all initial conditions are already Nash equilibria. Under the black dotted line, the initial wealth is smaller than $C^\star$ but the initial distribution does not satisfy Eq. (9). Therefore, in this region our model coincides with Bourlès, Bramoullé and Perez-Richet^12^ original model and there are no transfers to the public good. Other initial conditions will lead to a Nash equilibrium on the dark blue boundary, displaying some level of overconsumption and some level of inequality.

**Figure 3:**
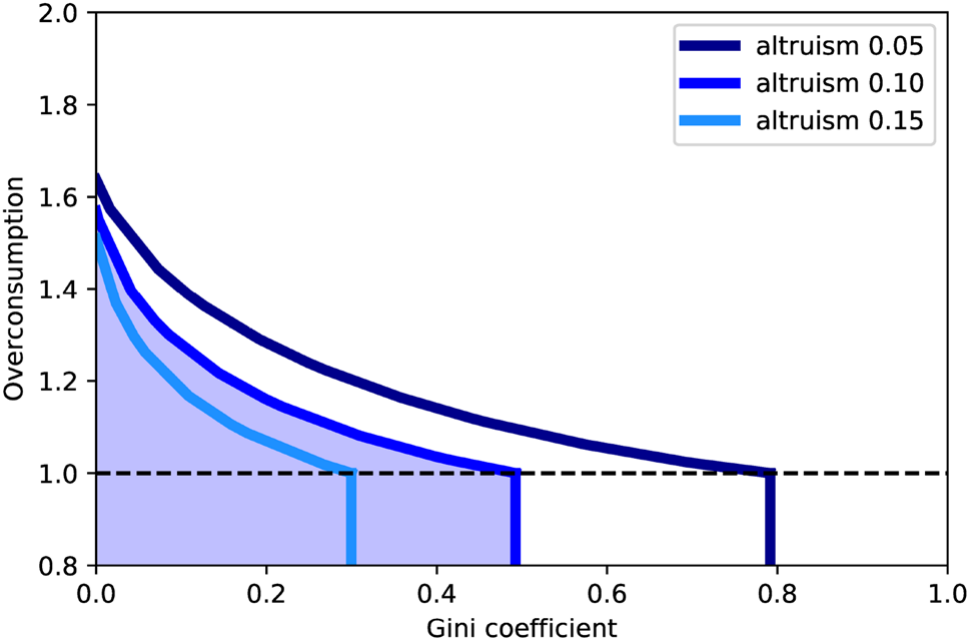
Impact of the altruism coefficient on the size of the set of Nash equilibria. Incrementing $\alpha$ shrinks the Nash equilibria region not only in terms of area; it also limits both the maximum overconsumption and the Gini coefficient.

Following this upper boundary of the Nash equilibria region (for a specific altruism coefficient) illustrates the trade-off between inequality and overconsumption: overconsumption can only be decreased at the cost of a higher Gini coefficient. However, increasing altruism shrinks the region of Nash equilibria, as shown in Fig. 3. Ultimately, for $\alpha = 1$, all Nash equilibria collapse to a single point: the social optimum. As expected, a society of fully connected, fully altruistic individuals behaves as recommended by the social planner solution (as all agents fully internalize the effect of their decision on the society at large). Therefore, for a fully connected network, increasing altruism simultaneously decreases inequality and overconsumption, allowing to “fill two needs with one deed”.

#### Regular trees

However, more realistic large-size social networks typically display far less connections than a fully connected network^14^. For example, based on empirical evidence, Dunbar^15,16^ proposed that a typical individual can maintain a stable relationship with at most 150 people. It is thus reasonable to consider an upper bound on the number of neighbours that a player can have, and even more so as we focus on networks with high altruism coefficient, i.e., strong ties between players.

We therefore investigate the behaviour of the game for a set of networks with a growing number of players, each player having at most *r* neighbours. As an example, we consider the case of 5-regular trees. The 5-regular tree of height 0 is a single player (the root). Then, from a 5-regular tree of height *n*, the 5-regular tree of height $n+1$ is obtained by adding 5 neighbours to every player of level *n*. This way, we can investigate the effect of the network size (number of players) while keeping the same structure (constant number of neighbours) and minimizing the increase of distances. More precisely, a tree-like structure allows for a fast size growth while keeping the distances among players as short as possible.

**Figure 4:**
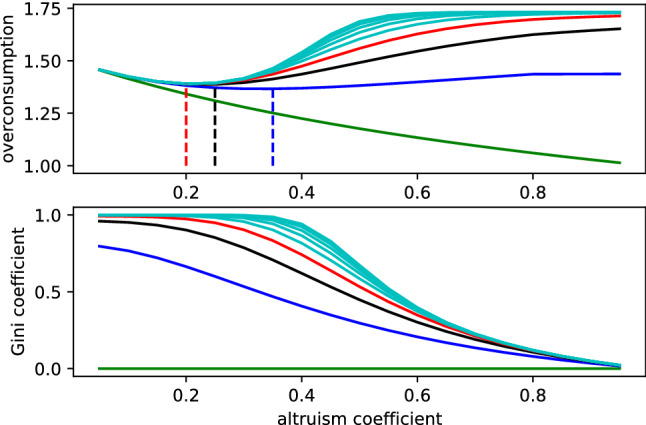
Overconsumption and Gini coefficient as a function of the altruism coefficient, for 5-regular trees of heights 1 (green), 2 (blue), 3 (black), 4 (red), and higher (cyan).

**Figure 5:**
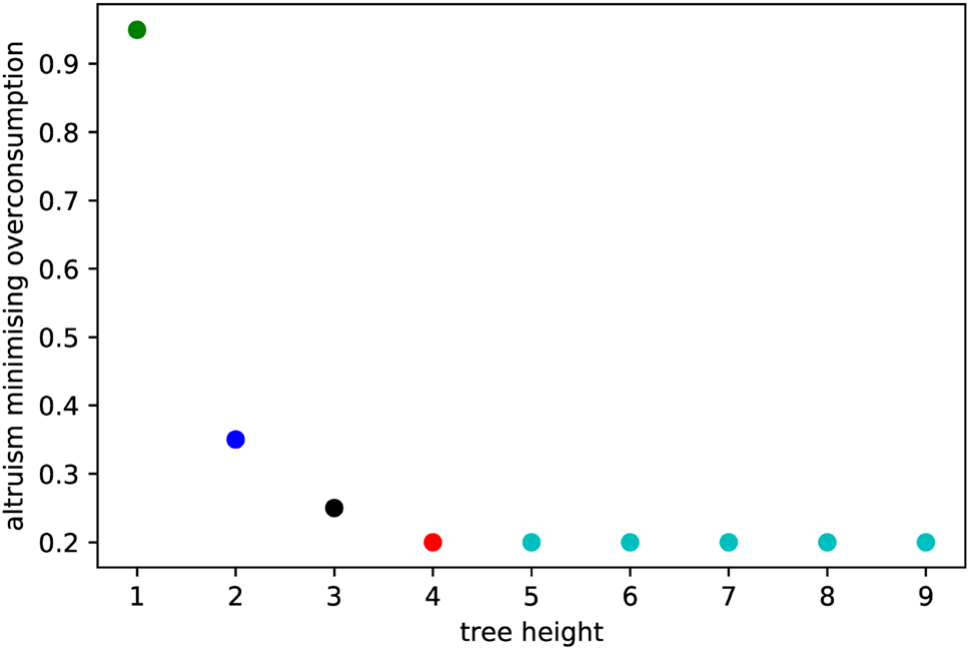
Critical values for the altruism coefficient with respect to tree height: the best altruism coefficient allowing for a simultaneous reduction of overconsumption and inequalities in the Nash equilibrium. Pushing $\alpha$ past this value increases overconsumption while still decreasing inequalities.

Due to computational constraints, the numerical simulations are done with constant total consumption *C* rather than a constant total initial budget $S_0+W$ as we do above for the fully connected case. It is still a relevant comparison since public good degradation is *in fine* generated by total consumption. Figures 4 and 5 show the computed Nash equilibria corresponding to a total consumption of 1000*N*, where the root player and its 5 (level one) neighbours are the richest players and inequalities between consecutive levels are set at the limit imposed by conditions (9). The minimum $S_0$ allowing such consumption distribution to be a Nash equilibrium has been used to compute the social optimum and therefore the overconsumption.

As displayed in Fig. 4, inequalities in 5-regular trees of any height decrease monotonically when altruism increases, even though the larger trees require higher altruism coefficient to reach a significant decrease in inequality. However, as soon as no player is linked to all other players (height 2 and higher), overconsumption starts increasing beyond some value of altruism. This effect is more marked and occurs at a lower altruism coefficient when the graph height increases: while overconsumption decreases up to an altruism coefficient of roughly 0.35 for a height of 2 (blue in Fig. 4), it starts increasing already at 0.20 for a height of 4 (red in Fig. 4). Note that in the latter case, such level of altruism is insufficient to decrease inequality significantly. This is the source of our paradox; increasing altruism can result in a degradation of the public good, the latter eventually entering in competition with inequality reduction.

A social planner trying to simultaneously minimize overconsumption and inequalities by selecting the optimal altruism coefficient will therefore face a trade-off between both objectives. The critical altruism coefficient diminishes for higher trees and, therefore, for larger networks, as shown in Fig. 5: indeed, in our simplified example, the critical altruism reaches its limit already for a 5-regular tree of height 4, as shown in Fig. 5. In other terms, if the altruism coefficient exceeds its critical value, any reduction in consumption inequality is obtained at the cost of an increase in overconsumption.

In sparse networks, the increase of altruism required to reduce inequalities results eventually in a higher degradation of the public good. Therefore, altruism in realistic networks leads to a trade-off between inequality reduction and public good preservation: there exists a tension between both objectives and increasing the coefficient of altruism is no longer a win-win strategy. We checked that this result is more generic than the examples provided above, by investing other classes of large sparse networks where players have a limited number of altruistic bonds. As detailed in the Supplementary Information, Nash Equilibria simulations for randomly generated sparse networks of type Erdös-Rényi^17^ (more precisely, Gilbert’s probabilistic version^18^), Watts-Strogatz^19^ and Barabási-Albert^20^ exhibit the same qualitative trade-off behavior between inequality reduction and public good conservation.

## Discussion

While our model is highly stylized, its simplicity allows to highlight the deep mechanisms at play. By evidencing that altruism can have opposing effects on inequalities and the deterioration of a public good, it sheds for example a new light on the negative correlation between inequalities and climate emissions evidenced in Fig. 1. However the inference is inevitably limited by the simplicity of the model and the functional form assumptions (in particular the utility functions).

We show that only in an idealized society where all players care about each other, increasing altruism results in a better protection of the public good. The mechanism at work for this positive output lies in the fact that the players with more altruistic bonds get a higher benefit from the improvement of the public good state and are therefore more likely to transfer to the public good themselves. In fact, at the theoretical limit $\alpha =1$, each player aims to maximize exactly the social planner’s utility function, which leads to the only situation where the paradox is avoided. This has profound implications for global common good issues such as climate change since, in practice, no individual player has the number of connections needed to fully enjoy the benefits provided by the public good. Consequently, inequality reduction takes place mostly through direct transfers to the neighbours, at the expense of public good provisioning.

With a number of neighbours bounded well below the number of players, which is usually the case for large networks, the players internalize less the global impact of consumption on the public good degradation as only the part enjoyed by their neighbours is accounted for in their social utility (even for very high levels of altruism). This phenomenon favours the redistribution mechanism, which increases overall consumption, and is exacerbated by high altruism coefficients, thus preventing the persistance of large clusters of disproportionately rich players, which are the ones transferring the most to the public good due to their lower marginal utility of consumption. Nonetheless, some altruism reduces overconsumption compared to non-altruistic networks. Altruism therefore contributes to some extent to the preservation of the public good, without however allowing to reach the social optimum.

By defining the network according to altruism, we consider that individuals are only altruistic to their relatives and do not care about strangers. As long as the network $\Gamma$ of players is connected, one could study an alternative situation, considering a complete network with non homogeneous altruism coefficients: if player *i* and player *j* are at distance *d* in the network, then their altruism coefficient is set to be $\alpha ^d$. It is worth mentioning that, for the Bramoullé, Bourlès and Perez-Richet model^12^ both games lead to the same equilibrium consumption. In the case of our model on a complete network, we have shown that increasing the altruism coefficient towards its upper limit of 1 approaches the optimal solution. Therefore, one would expect the alternative game to indeed provide better public good provisioning.

## Conclusion

Our simple model provides a rationale for the puzzling negative correlation between inequality reduction and $CO_2$ emissions: an increase in altruism tends to decrease consumption inequalities, but it can come at the cost of failing to preserve the common good (e.g. climate), especially at a global level. Indeed, large communities (e.g. countries) will have qualitatively different behaviours than small ones like families or villages, which usually have an underlying highly connected network structure. Inferring what happens in large settings from small scale experience might therefore lead to wrong conclusions, as is strikingly visible in our model output.

We have to dig deeper into the consequences of the different expressions of altruistic actions (direct transfers to other individuals and/or transfers to the public good) and better understand the tension between human development (say inequality reduction) and nature preservation (say reduction of $CO_2$ emissions) for all “realistic” network configurations. The existence of this trade-off certainly has implications for the current climate negotiations: it stresses the urgent need for formal cooperation among multiple stakeholders in order to achieve the previously agreed targets on reduction of $CO_2$ emissions and global warming.

Perhaps the solution lies in the reconciliation of the two mechanisms through which altruism finds its expression in our model namely, direct transfers to other individuals, or transfer to the common public good (say climate change mitigation). If the direct transfers to other individuals can be utilised in such a way to increase the wellbeing (in a broad sense) of these individuals, without contributing to climate change, then we would be able to achieve both human wellbeing and climate change mitigation simultaneously. This implies that we would have to deviate from the previous/current pathways to development in which improvement of human wellbeing necessarily implies a deterioration of the public good (climate), and resort to alternative pathways that promote human wellbeing through nature-preserving or even nature-enhancing solutions.

## Methods

All algorithms and simulations have been implemented in python using the *networkX* library^21^. The detailed algorithms and theoretical calculations can be found in the Supplementary Material.

## Supplementary Information

Supplementary Information.

## Data Availability

The scripts are available upon request to the first author.
