# Supplementary material for: Paradoxical effects of altruism on efforts to mitigate climate change
Source: Sci Rep. 2022 Jul 29;12:13072. doi: 10.1038/s41598-022-17535-y (PMC9338031; doi:10.1038/s41598-022-17535-y)
Supplement: Supplementary file 1 — Supplementary Information. [file 41598_2022_17535_MOESM1_ESM.pdf]

Supplementary Information

A. Fossas-Tenas, B.W. Ibelings, J. Kasparian, J. Krishnakumar, J. Laurent-Lucchetti

Paradoxical effects of altruism on efforts to mitigate climate change

Supplementary Methods

Social planner's optimal consumption

A social planner would maximise the sum of all players' social utilities, which can be written as

$$U_S(c_1, \ldots, c_N, W, S_0) = \sum_{i=1}^{N} u_i = \left(\sum_{i=1}^{N} (1 + \alpha d_i)\sqrt{c_i} \right) \sqrt{S_0 + W - \Sigma c_i - \frac{1}{2}(\Sigma c_i)^2}, \tag{S1}$$

where W is the (total) initial income, N the number of players, S_0 the initial state of the public good and d_i the degree of the i-th player in the network (the number of connections involving player i).

Note that U_S is a concave function of each c_i, hence it has a single maximum. This maximum is computed by finding the point at which all partial derivatives vanish:

$$\frac{\partial U_S}{\partial c_i} = \frac{(1 + \alpha d_i)\sqrt{S_0 + W - C - \frac{C^2}{2}}}{2\sqrt{c_i}} - \frac{(1 + C)\Sigma_{k=1}^{N} \left((1 + \alpha d_k)\sqrt{c_k} \right)}{2\sqrt{S_0 + W - C - \frac{C^2}{2}}} = 0, \tag{S2}$$

where C denotes $\Sigma_{i=1}^{N} c_i$ as in the main text. Suppose $c_i > 0$ for all i and $S_0 + W - C - \frac{C^2}{2} > 0$ (which is always the case for small enough c_i since initial conditions S_0 and W are strictly positive). Then, equation S2 is equivalent to

$$\frac{\sqrt{c_i}}{1 + \alpha d_i} = \frac{S_0 + W - C - \frac{C^2}{2}}{(1 + C)\Sigma_{k=1}^{N} \left((1 + \alpha d_k)\sqrt{c_k} \right)} \qquad \forall i. \tag{S3}$$

Since equation S3 holds for every player, we obtain the equilibrium distribution condition:

$$\frac{\sqrt{c_i}}{1 + \alpha d_i} = \frac{\sqrt{c_k}}{1 + \alpha d_k} \qquad \forall i, k \tag{S4}$$

which allows us to reduce our initial optimization problem to a single variable one, e.g. the optimization of c_1. Hence, by substitution we obtain:

$$C = \sum_{i=1}^{N} c_i = \frac{c_1}{(1+\alpha d_1)^2} \sum_{i=1}^{N} (1+\alpha d_i)^2 , \tag{S5}$$

$$\sum_{i=1}^{N} ((1+\alpha d_i)\sqrt{c_i}) = \frac{\sqrt{c_1}}{1+\alpha d_1} \sum_{i=1}^{N} (1+\alpha d_i)^2 . \tag{S6}$$

Let K denote $\sum\limits_{i=1}^{N} (1+\alpha d_i)^2$. Then, to solve equation S3 is equivalent to find the positive solution of:

$$-\frac{3K^2}{2(1+\alpha d_1)^3} c_1^2 - \frac{2K}{1+\alpha d_1} c_1 + (1+\alpha d_1)(S_0 + W) = 0, \tag{S7}$$

which is

$$c_1^\star = (1+\alpha d_1)^2 \frac{\sqrt{4 + 6(S_0+W)} - 2}{3K}. \tag{S8}$$

Thus, by equation S5

$$C^\star = \frac{\sqrt{4 + 6(S_0+W)} - 2}{3}. \tag{S9}$$

Note however that the total consumption cannot exceed the initial income W. Indeed, when the value of $C^\star$ is larger than the total initial income W, then the optimal consumption C_{opt} is capped to W:

$$C_{opt} = \min\{C^\star, W\}. \tag{S10}$$

Existence of Nash equilibria and corresponding equations

The social utility u_i is a concave function, thus first order equations are necessary and sufficient conditions for Nash equilibria. Let $W = \sum_{i=1}^{N} w_i$ be the total income, where w_i is player i's initial income. Let S_0 be the initial state of the public good, N the number of players, c_i the personal consumption of player i and $\mathcal{N}(i)$ denote the set of player i's neighbors. At equilibrium, the marginal utility, $\frac{\partial u_i}{\partial c_i}(c_1, \ldots, c_N, W, S_0)$, of the own consumption of each player i is bounded by the marginal utility of their transfer $t_{i,k}$ to their neighbour k:

$$\frac{\partial u_i}{\partial c_i}(c_1, \ldots, c_N, W, S_0) \geq \frac{\partial u_i}{\partial t_{i,k}}(c_1, \ldots, c_N, W, S_0) \qquad \forall k \in \mathcal{N}(i) \qquad \forall i \tag{S11}$$

and the equality holds when there the transfer from player i to player k indeed occurs.

Equivalently, the marginal utility of consumption for each player is bounded by the marginal utility of their transfer $t_{i,\mathrm{S}}$ to the public good,

$$\frac{\partial u_i}{\partial c_i}(c_1,\ldots,c_N,W,S_0) \geq \frac{\partial u_i}{\partial t_{i,\mathrm{S}}}(c_1,\ldots,c_N,W,S_0) \qquad \forall i \tag{S12}$$

and the equality holds when a transfer from player i to the public good actually takes place.

Since the initial personal income has either to be consumed, transferred to another player or transferred to the public good, it is clear that:

$$c_i = w_i - \sum_{k\in\mathcal{N}(i)} t_{i,k} - t_{i,S} + \sum_{k\in\mathcal{N}(i)} t_{k,i}. \tag{S13}$$

With some abuse of notation, all conditions can be written using only the terms $c_1,\ldots,c_N$, W and S_0. As in the main text, we denote the state of the public good $\mathcal{G} = \sqrt{S_0 + W - C - C^2/2}$. A simple calculation gives:

$$\frac{\partial u_{\mathrm{s},i}}{\partial c_i}(c_1,\ldots,c_N,W,S_0) \quad = \quad \frac{\cdot\mathcal{G}}{2\sqrt{c_i}} + \left(\sqrt{c_i} + \alpha \sum_{k\in\mathcal{N}(i)} \sqrt{c_k}\right)\cdot\frac{\partial\mathcal{G}}{\partial c_i} \tag{S14}$$

$$\frac{\partial u_{\mathrm{s},i}}{\partial c_k}(c_1,\ldots,c_N,W,S_0) \quad = \quad \frac{\alpha\cdot\mathcal{G}}{2\sqrt{c_k}} + \left(\sqrt{c_i} + \alpha \sum_{k\in\mathcal{N}(i)} \sqrt{c_k}\right)\cdot\frac{\partial\mathcal{G}}{\partial c_k} \tag{S15}$$

$$\frac{\partial u_{\mathrm{s},i}}{\partial t_{i,S}}(c_1,\ldots,c_N,W,S_0) \quad = \quad \left(\sqrt{c_i} + \alpha \sum_{k\in\mathcal{N}(i)} \sqrt{c_k}\right)\cdot\frac{\partial\mathcal{G}}{\partial t_{i,S}} \tag{S16}$$

All terms being well defined and non zero near the Nash equilibria, together with the fact that $\frac{\partial\mathcal{G}}{\partial c_i} = \frac{\partial\mathcal{G}}{\partial c_j}$, imply that equation S11 is equivalent to:

$$\sqrt{c_k} \geq \alpha\sqrt{c_i} \quad \forall k \in \mathcal{N}(i). \tag{S17}$$

Note that $W - C = T$, where $T = \sum_{i=1}^N t_{i,S}$. Thus, $\mathcal{G} = \sqrt{S_0 + T - \frac{C^2}{2}}$ and

$$\frac{\partial\mathcal{G}}{\partial c_i} \quad = \quad \frac{-C}{2\sqrt{S_0 + T - \frac{C^2}{2}}} \tag{S18}$$

$$\frac{\partial\mathcal{G}}{\partial t_{i,S}} \quad = \quad \frac{\sqrt{c_i} + \alpha\sum_{k\in\mathcal{N}(i)}\sqrt{c_k}}{2\sqrt{S_0 + T - \frac{C^2}{2}}} \tag{S19}$$

Hence, equation S12 is equivalent to

$$S_0 + W - C - \frac{C^2}{2} \geq \left(c_i + \sum_{k\in\mathcal{N}(i)} \alpha\sqrt{c_i c_k}\right)(1 + C)\,. \tag{S20}$$

Since these are true for each player, we can agregate them all to obtain the following Nash equilibria condition:

$$\frac{S_0 + W - C - \frac{C^2}{2}}{1 + C} \geq \max_{i \leq N} \left\{ c_i + \sum_{k \in \mathcal{N}(i)} \alpha \sqrt{c_i c_k} \right\}. \tag{S21}$$

Complete network computations

For complete networks (*i.e.* when every pair of players is connected), equation S17 limits the consumption inequality at the Nash equilibrium to a factor of α^2. As a consequence, for a network containing one single rich player, the maximal Gini coefficient compatible with a Nash equilibrium corresponds to the situation where the rich player consumes c_r and all other players consume exactly $\alpha^2 c_\mathrm{r}$ (which corresponds to the red dot on figure 2 of the main text). It is worth noting that there is no overconsumption in the corresponding Nash equilibrium.

We consider a fully connected network featuring 1 rich player, with income w_r and 99 poor ones (with income w_p) and we assume that initial conditions S_0, $W = w_\mathrm{r} + 99w_\mathrm{p}$ and α are given. In this case, the binding term for equation S21 is the one corresponding to the richest player.

If equation S21 is fulfilled, i.e. if no transfer to the public good is needed, the Nash equilibrium depends on the need of transfers between players as stated by equation S11. If equation S11 is fulfilled, the initial income distribution together with the initial S_0 is already a Nash equilbrium, which corresponds to the blue region of the graph. Conversely, if equation S11 is not fulfilled, transfers only occur from the rich player to the poorer ones, i.e. the initial conditions belong to the region under the dotted black line of figure 2 of the main text. In this case, the Nash equilibrium is the point in the vertical blue line featuring $W = (1 + 99\alpha^2)c_r$ and $c_p = \alpha^2 c_r$.

When transfer to the public good is needed, it will be performed by the richest player alone up to an amount of $w_r - w_p$, i.e., as long as

$$\frac{S_0 + W - 100w_\mathrm{p} - \frac{(100w_\mathrm{p})^2}{2}}{1 + 100w_\mathrm{p}} \geq w_\mathrm{p}(1 + 99\alpha), \tag{S22}$$

When equation S22 is fulfilled, the Nash equilibrium reached depends on the value w_p. Two different cases need to be considered:

1. Either the solution to the equation:

$$\frac{S_0 + W - 99w_p - c_r - \frac{(99w_p + c_r)^2}{2}}{1 + 99w_p + c_r} = c_r + 99\alpha\sqrt{c_r w_p} \tag{S23}$$

   satisfies $w_p \geq \alpha^2 c_r$, so $c_p = w_p$, and c_r is obtained this way. This corresponds to the initial conditions on the purple region of figure 2 and yields a Nash equilibrium in the blue line between the green and the red dots.

2. Or $c_p = \alpha^2 c_r$, where c_r is the solution to the equation:

$$\frac{S_0 + W - (1+99\alpha^2)c_r - \frac{((1+99\alpha^2)c_r)^2}{2}}{1+(1+99\alpha^2)c_r} = c_r(1+99\alpha^2). \tag{S24}$$

   This corresponds to the initial conditions on the red region of figure 2 and yields as Nash equilibrium the red dot.

Finally, when further transfer is required, i.e., if equation S22 is not fulfilled, the rest of the transfer will be equally distributed among all players and the Nash equilibrium reached will be the one consisting in equally distributed consumption. In this case, at the Nash equilibrium the Gini coefficient of the consumption distribution is $g_{\mathrm{Ne}} = 0$ and the corresponding total consumption $C_{Ne} = 100c$ is obtained by solving the equation:

$$\frac{S_0 + W - 100c - \frac{(100c)^2}{2}}{1+100c} = c(1+99\alpha). \tag{S25}$$

This situation corresponds to initial conditions in the green region of the figure 2 on the main text, and the Nash equilibrium at the green dot.

Once the consumption distribution of the corresponding Nash equilibrium is computed, one can easily compute its total, C_{Ne}, as well as the corresponding Gini coefficient, g_{Ne}. Finally, as C_{opt} only depends on $S_0 + W$ and W, overconsumption is simply computed as $C_{\mathrm{Ne}}/C_{\mathrm{opt}}$.

The Nash equilibria boundaries on figures 2 and 3 of the main text where obtained by implementing the above algorithm with 100 players, $S_0 + W = 10^6$, $W \in \{1200, 2500, 500\}$ and the fraction of W dedicated to w_{r} varying from 1 to 0.1 by decreasing steps of 0.1.

The dotted lines marking the boundaries in the phase diagram (see figure 2 of the main text) were obtained by numerically solving the overlap of the different cases described above.

The green dotted line was obtained by first numerically solving equation S25 for c, and then calculating a series of 25 points by incrementing the value of $W/C^\star$, therefore obtaining $w_{\mathrm{r}} = W - 99c$. Similarly, for the red dotted line, we first calculated the corresponding Nash equilibrium without overconsumption by solving equation S24 for c_{r}. Then, a total of 25 points on the red dotted line were found by progressively incrementing $W/C^\star$, finally yielding $w_{\mathrm{r}} = W - 99\alpha^2 c_{\mathrm{r}}$. Finally, the black dotted line corresponds to setting $W = C^\star$ and $w_{\mathrm{r}} > \dfrac{W}{1+99\alpha^2}$.

Supplementary results

Asymptotic behaviour of the social optimum

The social planner's utilities, the optimal consumption, and the final state of the public good all increase when increasing the total initial budget $W + S_0$. However, they do not increase at the same rate.

Indeed, the total consumption is asymptotically proportional to the square root of the initial budget, see equation 9 of the main text, while the final condition of the public good under optimum consumption is linear on the initial budget:

$$S^\star = S_0 + W - C^\star - \frac{(C^\star)^2}{2} \tag{S26}$$

$$= S_0 + W - \frac{-2+\sqrt{4+6(S_0+W)}}{3} - \frac{\left(\frac{-2+\sqrt{4+6(S_0+W)}}{3}\right)^2}{2} \tag{S27}$$

$$\asymp \frac{2}{3}(S_0+W) \tag{S28}$$

and social utility is asymptotically proportional to the power $\frac{3}{4}$ of the total budget:

$$U_S(c_1^\star,\ldots,c_N^\star,W,S_0) = \left(\frac{K\sqrt{c_1^\star}}{1+\alpha d_1}\right)\sqrt{S_0+W-C^\star-\frac{(C^\star)^2}{2}} \tag{S29}$$

$$\asymp \frac{\sqrt{2K}}{3}\left(S_0+W\right)^{\frac{3}{4}}. \tag{S30}$$

Hence, at the optimum, in relative the initial income devoted to consumption rises slower than the share allocated to the public good. This is exemplified in figure S1. For an initial income $W = 10^4$, the optimal total utility of 733 is reached for $C = 81$. Increasing the initial income by 10% only rises the optimal consumption to $C = 85$, i.e. an increase of 5%, while the corresponding total utility reaches 788, i.e. an increase of 7.5%.

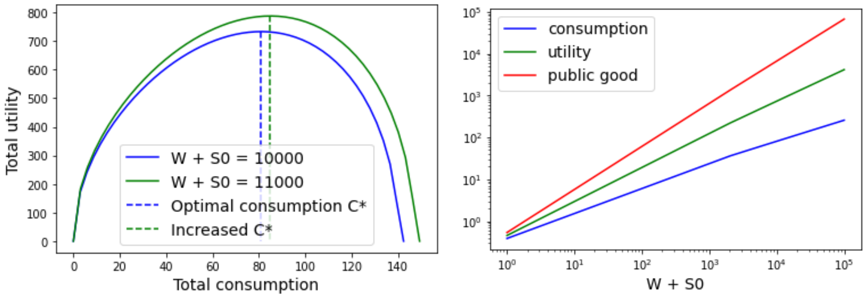

Figure S1: (a) Optimization of total utility for two different values of $W + S_0$. The optimal consumption $C^\star$ increases slower than $S_0 + W$. (b) Optimal consumption, total utility and public good state at the social optimum for increasing initial income.

Finally, we note that the quadratic degradation of the public good with consumption sets a natural upper bound to the latter: in order to keep a nonzero

utility, players need to ensure

$$S_0 + W - C - C^2/2 > 0. \tag{S31}$$

Thus $C \leq \sqrt{1 + 2(S_0 + W)} - 1$. Hence, for a sufficiently large initial amount of $S_0 + W$, the upper bound of C and $C^\star$ are, respectively, well approximated by $\sqrt{2(S_0 + W)}$ and $\frac{\sqrt{6(S_0+W)}}{3}$, see equation 9 of the main text. Overconsumption, $\frac{C}{C^\star}$, tends therefore to $\sqrt{3}$ for large initial $S_0 + W$. This value is also approached in all our simulations when players are all totally disconnected from each other and the total income is such that some transfer to the public good is needed. This *full overconsumption regime* in an egoistic setup behaves as the standard tragedy of the commons.

Alternative social optimum, based on personal utility

Since the social utility can be decomposed as a personal utility term plus the discounted personal utilities of the neighbours:

$$u_i(c_1, \dots, W, S_0) = \sqrt{c_i}\sqrt{S_0 + W - C - \frac{C^2}{2}} + \alpha \sum_{k \in \mathcal{N}(i)} \sqrt{c_k}\sqrt{S_0 + W - C - \frac{C^2}{2}}, \tag{S32}$$

the social planner could maximize the sum of the personal utilities instead of the sum of the social utilities:

$$U_{\mathrm{P}} = \left(\sum_{i=1}^{N} \sqrt{c_i}\right)\sqrt{S_0 + W - C - \frac{C^2}{2}}. \tag{S33}$$

In this case, for a given amount of total consumption C, considering the concavity of the utility function, the Cauchy-Schwarz inequality implies that U_{P} is maximized by equally distributing C among all players. Hence, when optimizing U_{P}, the social planner ends up optimizing the following function:

$$U_{\mathrm{P}}(C) = \sqrt{N}\sqrt{C}\sqrt{S_0 + W - C - \frac{C^2}{2}}. \tag{S34}$$

Interestingly, the optimum is reached at the same level of consumption $C = C^\star$ as when considering the sum of social utilities. However the distribution of C^* among all players are different, except for regular graphs (i.e. graphs where all players have the same number of neighbors), where the function S34 is clearly a multiple of the function U_S of the main text.

Influence of the network topology

In order to assess the generality of the trade-off between inequality reduction and public good conservation with regard to the network topology, we have

computed several Nash equilibria for the 5-regular tree of height 4, a more connected and regular variant of such a tree and three networks with the same connectivity and the same number of vertices obtained using three classical random network models: Erdös-Rényi networks [1] (using Gilbert's probabilistic version [2]), Watts-Strogatz networks [3] and Barabási-Albert networks [4]. We have chosen height 4 because it already has a low critical altruism parameter in the Pareto front (see figure 5 of the main text) and the algorithms we use in our simulations are still manageable in time and storage space in a desktop computer with the corresponding number of players.

The Erdös-Rényi model was the first introduced in [1] and is the most *natural* definition of a random network. Indeed, in an Erdos-Rényi-Gilbert network, each of the possible connections (in our particular case $\frac{781 \cdot 780}{2}$) is present with the same probability (in our particular case $\frac{6}{781}$), and therefore absent with probability $1 - \frac{6}{781}$. With this parameter choice, the network obtained has a main connected component and at most a few other small components (less than five components of one or two vertices). Since we want to deal with connected networks, if the network obtained with the random generator is not connected, we add a connection from each of the connected components to the main connected component.

The Watts-Strogatz model was introduced as a variation from the Erdös-Rényi to better mimick the fact that real sociological networks usually have a higher clustering coefficient than the one obtained with the Erdös-Rényi model, i.e. neighbours of the same player are more connected among them than what would be expected by random connections. For the Watts-Strogatz network, with our parameter choice, the generator model starts with a cycle of length 781 where each player is connected to his 6 nearest players (3 turning left, three turning right). Then, each connection is randomly flipped (changed to a connection with a randomly chosen vertex) with probability $\frac{1}{4}$. This probability ensures still a high clustering coefficient while already having the small world property.

The Barabási-Albert model was introduced to better approach the degree distribution of real networks. For the Barabási-Albert network, with our parameter choice, the generator model starts with a star of 7 vertices and then grows by one player at each step. Each new player is connected to exactly 6 pre-existing players with a probability proportional to their degree at that step.

Like for the tree networks in the main text, we set the total consumption (here, $C = 5 \cdot 10^8$) rather than the total income and we look for Nash equilibria all having the same amount of total consumption C. To find the Nash equilibria, we first choose the set of richest players. We then set the consumption of their neighbours at the α^2 relative inequality with respect to them, see equation S17, the neighbours of their neighbours at α^4, and we use iteratively equation S17 until every player has a non-zero consumption (the process ends because we consider only connected networks). We normalize the resulting individual con-

sumptions to sum up to C. Finally, we set S_0 based on equation S21

$$S_0 = \max_{i \leq N} \left\{ c_i + \sum_{k \in \mathcal{N}(i)} \alpha \sqrt{c_i c_k} \right\} \cdot (1 + C) + C + \frac{C^2}{2} - W. \qquad \text{(S35)}$$

We first compute 25 different Nash equilibria for several consumption distributions on a 5-regular tree of height 4(and hence 781 players), whose corresponding Gini coefficient and overconsumption are represented with green squares in figure S3:

- Equidistributed consumption.
- A single rich: a total of 5 different configurations depending on the height of the rich player (from the root to the leaves).
- A single poor: a total of 5 different configurations depending on the height of the poor player.
- A rich cluster constructed as a single player and all his neighbours: again a total of 5 different configurations.
- A rich cluster of all players at distance 2 or less from a single player: also 5 different configurations.
- 25 randomly chosen rich players: 4 such configurations.

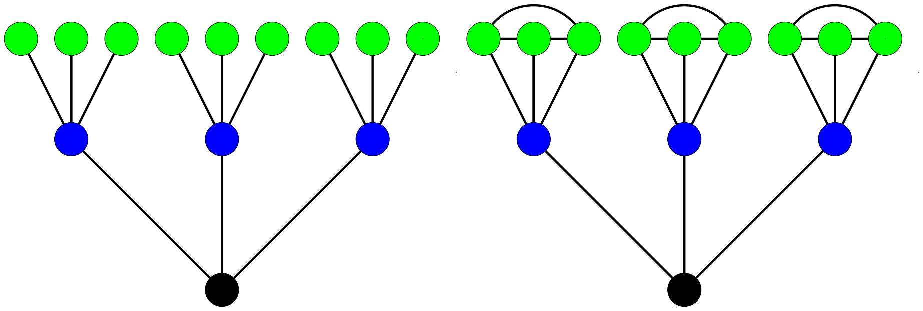

Figure S2: A 3-regular tree of height 2 (the root in black, the vertices on level one in blue and the vertices on level two in green) and its modified version.

We then slightly modify the tree network to obtain a regular network where all players have 5 neighbours. To do so, we connect the height 4 players, which have a single connection, in groups of 5 fully connected neighbours. This process is shown in figure S2 for a 3-regular tree of height two. We compute the same 25 Nash equilibria as for the original tree. This modification of the tree does not affect the overconsumption and Gini coefficients of the Nash equilibria, as

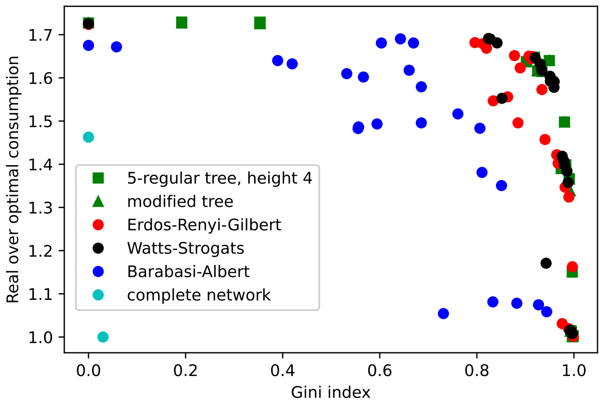

Figure S3: The Gini coefficient and overconsumption for the Nash equilibria in different sparse networks with the same number of players and similar connectivity.

shown by the fact that the corresponding green squares triangles are almost superposed in figure S3.

We use the random generators mentioned above (Erdös-Rényi [1], Watts-Strogatz [3], and Barabási-Albert [4] models) to obtain three different (connected) networks with 781 players and mean degree of 6: the parameters for the random generators have been chosen to ensure having the same number of players and the same mean degree as in the modified tree case, in order to obtain networks with comparable density. One should nevertheless remark that with only 781 players, the differences between the three models are less significant than what they would be for larger networks. Finally, we also compute the equidistributed Nash equilibrium and a single rich Nash equilibrium for the complete network with 781 players and the same total consumption. The results are shown in figure S3.

The trade-off between reducing inequalities and reducing overconsumption in all investigated non-complete networks is clear from figure S3. Solutions minimising both overconsumption and inequalities would be located in the lower-left corner of the graph, which is not observed from our simulations except for the trivial case of the complete graph. The fact that this behaviour is shared between networks covering a wide variety of situations illustrates its generality.

Code availability

All algorithms and simulations have been implemented in python using the *networkX* library [5]. The scripts are available upon request to the first author[1].

References

[1] P. Erdös and A. Rényi. On random graphs. *Publ. Math.*, 6:290–297, 1959.

[2] E.N. Gilbert. Random graphs. *Ann. Math. Stat.*, 30:1141–1144, 1959.

[3] D. Watts and S. Strogatz. Collective dynamics of 'small-world' networks. *Nature*, 393:440–442, 1998.

[4] A.L. Barabási and R. Albert. Emergence of scaling in random networks. *Science*, 286:509–512, 1999.

[5] Networkx python library. *https://networkx.org*.

[1] ariadna.fossastenas@gmail.com
